# Environmental and Occupational Risk Factors for Retinoblastoma

**DOI:** 10.1016/j.focus.2026.100534

**Published:** 2026-07-21

**Authors:** Rebecca A. Bowen, Therese S. Woodring, Eric J. Monroe, Sheryl A. Bedno

**Affiliations:** 1 Wisconsin Department of Health Services, Waukesha, Wisconsin; 2 Division of Pediatric Hematology, Oncology, Transplant & Cellular Therapy, University of Wisconsin-Madison, Madison, Wisconsin; 3 Pediatric Interventional Radiology, Department of Radiology, University of Wisconsin-Madison, Madison, Wisconsin

**Keywords:** Retinoblastoma, environmental, occupational, risk factors, cancer cluster

## Abstract

**Introduction:**

The Wisconsin Department of Health Services received notice of an unusual number of cases of retinoblastoma in a small geographic area. Retinoblastoma is a rare pediatric eye cancer that strikes children aged <5 years. The reporters of these cases were concerned that they may be part of a cancer cluster. Cancer clusters are unusual numbers of cases in a defined geographic area over a defined period of time. They are believed to be caused by shared environmental or occupational risk factors. It is not known to what extent these risk factors play a role in retinoblastoma incidence.

**Methods:**

As part of state standard operating procedures for cancer cluster investigations, Wisconsin Department of Health Services performed a scan of the scientific literature to determine what is known about environmental or occupational risk factors in an attempt to consider the likelihood that the observed cases could be attributed to a shared community factor. Throughout this process, over 1,200 abstracts were scanned, and 41 papers were read in depth. This narrative review is the result of that literature review surrounding such risk factors and retinoblastoma incidence.

**Results:**

Although much is known about the genetic abnormalities that result in tumor development, little is known about environmental and occupational risk factors and the potential roles they play in retinoblastoma development. Few studies have been found, and those that are published are often hampered by small numbers owing to the rarity of this cancer. Although some studies have shown potential correlations between exposures and incidence, these results need to be corroborated by other studies. This review unfortunately did not provide direction for continuing an inquiry into the potential cluster of cases, and the cancer cluster investigation that precipitated this review was closed.

**Discussion:**

Like all rare cancers, progress toward understanding the root cause(s) of retinoblastoma is difficult because of the small number of cases per year. Still, future studies should continue to add to the body of knowledge about environmental and occupational risk factors for this pediatric cancer, so that the weight of collective studies can inform future prevention and treatment efforts.

## HIGHLIGHTS

• A potential cancer cluster of retinoblastoma cases was identified in Wisconsin.
• This review looks at potential risk factors that could lead to clustering.
• It focuses on environmental and occupational risk factors.
• Studies are hampered by low numbers of retinoblastoma cases.
• Some studies showed potential associations but need to be corroborated.

## INTRODUCTION

Retinoblastoma is a rare pediatric eye cancer that is typically diagnosed before the age of 2 years.^1^ The Wisconsin Department of Health Services (DHS) was contacted regarding several cases of retinoblastoma in a community. There was concern that these cases might be part of a cancer cluster. Cancer clusters are unusual numbers of cases of a specific type of cancer in a defined geographic area over a defined period of time. They are believed to be related to shared factors in the environment or to occupational exposures.

Retinoblastomas comprise 3% of all childhood cancers and 6.1% of childhood cancers among aged <5 years.^2^ They are rare cancers, with only 200–300 cases diagnosed per year in the U.S.^3^ Retinoblastomas are categorized on the basis of whether 1 eye is affected (unilateral retinoblastoma) versus both eyes (bilateral retinoblastoma) versus both eyes and some additional brain involvement (trilateral retinoblastoma).^3^ Retinoblastoma can alternately be described as familial/heritable or sporadic on the basis of whether the defective retinoblastoma gene (*RB1*) is inherited or newly altered.^4^ If a defective copy of *RB1* is inherited, the risk of retinoblastoma developing is 90%.^3^

As part of the standard operating procedures within DHS, a scan of the scientific literature was conducted to learn what is known about environmental or occupational risk factors for this cancer type and to direct further steps in a cancer cluster investigation. DHS found that although many reviews exist concerning the genetics of retinoblastoma, about which much is known,^3^^,^^5^, ^6^, ^7^ none were found discussing environmental or occupational risk factors for the disease. This is not surprising owing to the rarity of this cancer. Small annual case numbers mean that most case-control studies are underpowered to detect differences in exposures. Most studies have focused on maternal and paternal behavioral risk factors, such as diet,^8^, ^9^, ^10^ age,^11^, ^12^, ^13^, ^14^, ^15^, ^16^, ^17^, ^18^ health,^19^^,^^20^ and smoking.^21^, ^22^, ^23^, ^24^, ^25^ Few have examined environmental or occupational risk factors, often offering conflicting results.

To DHS’s knowledge, no review exists concerning known environmental or occupational risk factors and retinoblastoma. To fill this gap, the available literature was reviewed with the aim of assisting other public health and medical professionals who are examining environmental and occupational concerns around retinoblastoma etiology and compiling what is known about the epidemiology of retinoblastoma, so as to inform areas where future research may be fruitful. This narrative review is the product of that work.

## METHODS

DHS’s goal was to evaluate potential environmental or occupational risk factors for retinoblastoma as part of a suspected cancer cluster investigation. To this end, a literature review was conducted in December 2024 with the intention of producing a narrative review on this topic. Using PubMed, the following search terms were used: *(risk factors)* OR *environmental* OR *occupational* AND *retinoblastoma*. This search resulted in 1,605 papers. When the results were restricted to papers in English, there were 1,533. When nonhuman papers were excluded, there were 1,200 papers. Abstracts were evaluated for relevance, resulting in 41 papers being reviewed (Figure 1). Papers focusing on nonenvironmental or occupational factors, such as maternal health, were excluded. Papers that met the inclusion criteria were divided into environmental or occupational categories. Similar exposures, such as those to pesticides, were then grouped. No artificial intelligence was used in this project. The results of this literature review are presented below. This work was performed as part of regular public health activities and was exempt from IRB approval. This research did not receive any specific grant from funding agencies in the public, commercial, or not-for-profit sectors.

**Figure 1:**
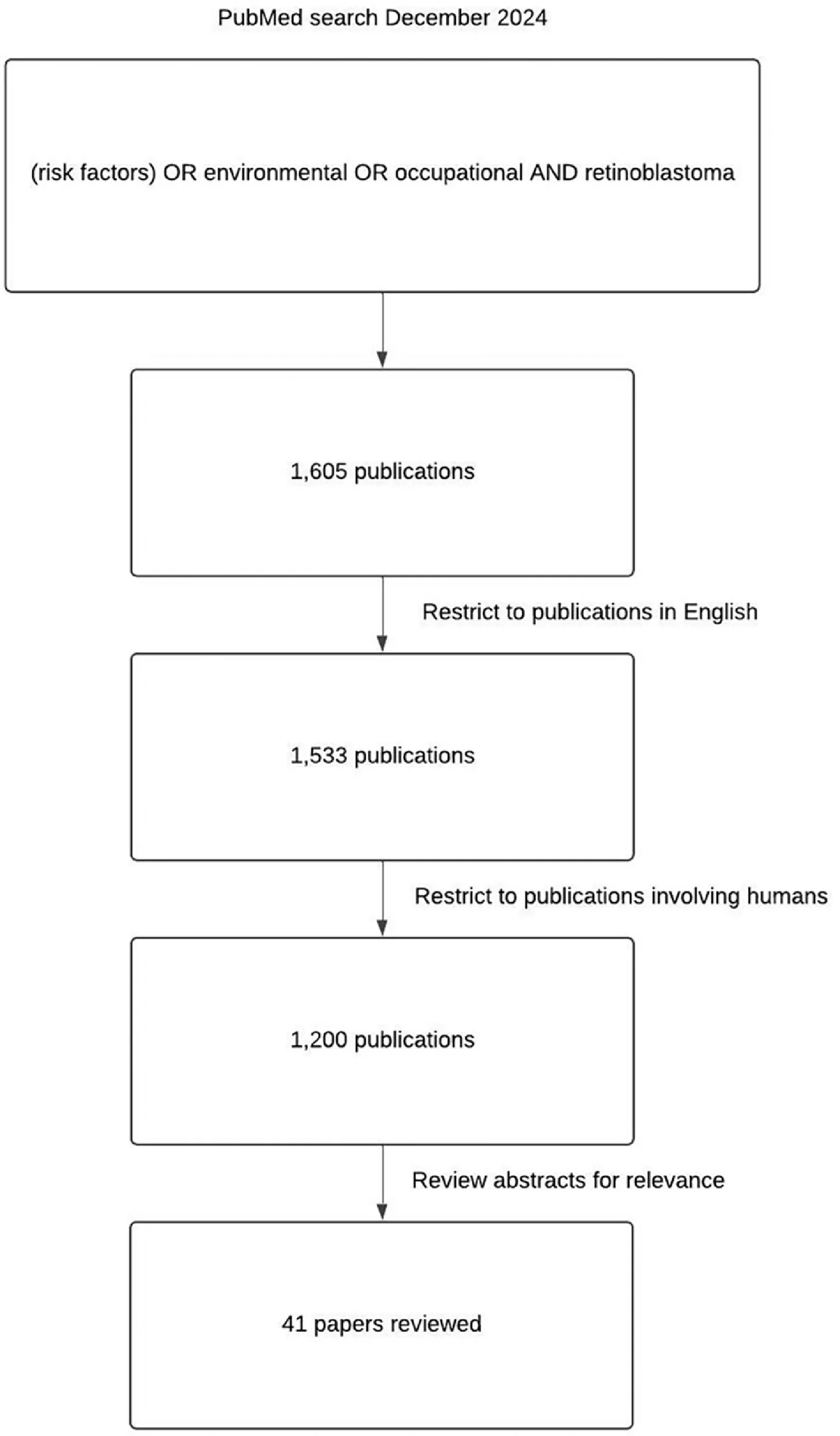
Search strategy.

### Pathogenesis and Pathophysiology

Retinoblastoma is an embryonal tumor that is typically diagnosed within the first 2 years of life.^3^ Without treatment, retinoblastoma will progress to visible bulging of the eye with a median period of 13.7 months, systemic metastasis 2.6 months later, and death at 2.0 months after metastasis begins.^26^ If the tumor has spread beyond the eye, it is fatal in 50%–85% of cases.^27^ If untreated, 100% of children die within 48 months of diagnosis.^26^ If detected early, retinoblastoma is curable in over 90% of patients, although enucleation (removal of the eyeball) might be required.^3^

Retinoblastoma develops as a result of mutations to the retinoblastoma gene (*RB1*). The cell of origin is believed to be a cone-cell precursor that develops in the middle layer of the retina, outside the outer layer where normal cone cells are located.^5^ *RB1* is involved in cell cycle regulation at the G1–S checkpoint.^3^ Upon inactivation through phosphorylation, it no longer represses the cell cycle and allows dysregulated cell division to occur.^3^

The mutations that result in *RB1* inactivation are diverse, ranging from point or frameshift mutations to large chromosomal deletions that involve not only *RB1* but also adjacent genes.^4^ In sequencing studies, over 50% of these mutations have been seen only once.^3^ Although loss of *RB1* function is necessary for tumorigenesis to occur, it is not sufficient and requires a further push.^28^ What this push consists of is currently unknown but may involve genetic imprinting, a form of epigenetic gene regulation that involves specific alterations in gene expression on the basis of maternal or paternal inheritance and has been shown to be involved in the development of retinoblastoma.^29^ The factors that lead to the mutation of *RB1* could be different from those that turn a mutated cell into a growing tumor.

### Retinoblastoma Incidence

There are between 200 and 300 cases of retinoblastoma annually in the U.S.^3^ Although the annual case rate is not reported by the National Cancer Institute, it has been reported in the literature to be between 3–4 per 1,000,000^30^ and 11.8 per 1,000,000^31^ children aged 0–4 years. This rate has been stable over the past 40 years,^31^^,^^32^ Wisconsin, for example, averages fewer than 6 cases per year and had a rate of 4.5 per 1,000,000 children aged ≤19 years as of 2020, the most recent year for which data are currently available. Although the incidence of retinoblastoma is the same for males and females,^3^^,^^31^ boys tend to be diagnosed more frequently, especially in developing nations.^3^ It is believed that this may be related to higher rates of healthcare-seeking behavior for sons than for daughters (incidence rate ratio=1.05 [boy to girl] [95% CI=1.02, 1.09]).^33^

### Environmental Risk Factors

Many groups have examined whether environmental factors could lead to an increased risk for either the mutations behind retinoblastoma development or tumor promotion (Table 1). Pesticide exposure, in particular, is an environmental factor that has been under scrutiny. A case-control study for both bilateral and unilateral retinoblastoma found that the use of a specific chemical agent to kill insects or diseases on the lawn was associated with an increased risk of developing unilateral retinoblastoma but not bilateral retinoblastoma (OR=2.8, 95% CI=1.1, 6.7).^34^ However, the increased risk was the same regardless of the type of product used (sprays versus other types), the location of the product (inside or outside the home), and when during pregnancy they were used. The absence of these variables in the analysis makes it difficult to deduce an exposure pathway that could be linked to tumor development.

**Table 1:** Select Studies Investigating Environmental Exposures and Retinoblastoma Incidence

Exposure	Study, year, location	Study design and sample size	Cofounders	Findings
Pesticides	Awounou et al.,^36^ 2024, France	Case-control; 403 retinoblastoma cases; 5,000 controls	Age	No increased risk with residential proximity within 1,000 m of vineyards (OR=1.1, 95% CI=0.8, 1.6)
	Thompson et al.,^35^ 2022, California	Population-based case-control; 335 cases (221 unilateral and 114 bilateral); and 123,166 controls	Birth year, child's sex, maternal age, neighborhood SES, maternal race/ethnicity	Increased risk of unilateral retinoblastoma associated with exposures to acephate (OR=1.70, 95% CI=1.20, 2.41) and bromacil (OR=1.87, 95% CI=1.07, 3.26); nonsignificant trend toward increased risk for retinoblastoma (all types combined) with exposure to pymetrozine (OR=1.45, 95% CI=1.00, 2.08) and kresoxim-methyl (OR=1.60, 95% CI=1.00, 2.56)
	Omidakhsh et al.,^34^ 2017, U.S.	Case-control, 282 cases (186 unilateral, 96 bilateral), and 148 controls	Mother's race, mother's employment status, whether the father was living in the home at the start of the pregnancy, mother's age	Increased risk of unilateral retinoblastoma associated with parental insecticide use (OR=2.8, 95% CI=1.1, 6.7), no difference between use inside and outside the home; nonsignificant trend toward increased risk of unilateral disease with use of professional lawn or landscaping services (OR=2.8, 95% CI=1.0, 8.2)
PFAS	Chen et al.,^39^ 2024, California	Case-control, 501 cases, and 899 controls		PFOS: Increased risk of unilateral retinoblastoma (OR=1.42, 95% CI=1.03, 1.97); nonsignificant trend toward increased risk of overall retinoblastoma (OR=1.29, 95% CI=1.00, 1.67)
Air pollution	Chen et al.,^46^ 2024, California	Case-control; 29 retinoblastoma cases; and 283,620 controls	Maternal age, mother's race and ethnicity, method of payment for prenatal care, a proxy for SES, a neighborhood SES-index variable, residence in an urban/rural environment	No increased risk for retinoblastoma with exposure to lead, nickel, or cobalt in air
	Kumar et al.,^41^ 2018, Texas	Population-based case control; 121 cases (89 unilateral and 32 bilateral); and 2,855 controls	Birth weight, gestational age, maternal age at delivery, maternal race/ethnicity, area-level poverty	Increased risk for unilateral retinoblastoma with every kilometer closer to a major roadway that the mother lived (AOR=2.7, 95% CI=1.28, 5.15)
	Garcia-Perez et al.,^42^ 2016, Spain	Population-based case-control, 139 cases, and 834 controls	Year of birth, sex, autonomous region of residence, percentage of total crop surface, percentage of illiteracy, percentage of unemployed, SES	No increased risk for retinoblastoma associated with living in urban areas
	Heck et al.,^45^ 2015, California	Population-based case-control, 103 cases, and 30,601 controls	Maternal race/ethnicity and nativity, paternal age, year of birth, SES indicator, method of payment for prenatal care	Increased risk for retinoblastoma with higher estimated perinatal exposure to styrene (OR=1.64, 95% CI=1.12, 2.39), ortho-xylene (OR=1.64, 95% CI=1.03, 2.61), toluene (OR=1.79, 95% CI=1.10, 2.92), chloroform (OR=1.53, 95% CI=1.10, 2.12), formaldehyde (OR=1.42, 95% CI=1.08, 1.88), total chromium (OR=1.34, 95% CI=1.01, 1.77), and acetaldehyde (OR=1.62, 95% CI=1.06, 2.48)
	Heck et al.,^40^ 2013, California	Population-based case-control; 254 cases; and 80,224 controls	Mother's and father's race/ethnicity, mother's years of education, mother's country of birth, parity, method of payment for prenatal care, neighborhood SES	Increased risk of bilateral retinoblastoma with higher modeled average traffic exposure during pregnancy (OR=1.10, 95% CI=1.01, 1.20) and during the first year of the child's life (OR=1.11, 95% CI=1.02, 1.22); inconsistent elevated risk for PM_2.5_ exposure between Model 1 and Model 1; nonsignificant trend toward increased risk for traffic density within 500 m of the residence (OR=1.05, 95% CI=0.97, 1.13)
	Ghosh et al.,^44^ 2013, California	Case-control; 87 cases of bilateral retinoblastoma; and 80,658 controls	Maternal age, race/ethnicity, educational level, parity, prenatal care insurance type, socioeconomic score quintile	Increased risk of bilateral retinoblastoma with third-trimester increases in nitric oxide pollution (OR=1.15, 95% CI=1.01, 1.31)
				
Proximity to industry	Garcia-Perez et al.,^42^ 2016, Spain	Population-based case-control; 139 cases; and 834 controls	Year of birth, sex, autonomous region of residence, percentage of total crop surface, percentage of illiteracy, percentage of unemployed, SES	Increased risk of retinoblastoma with proximity to glass and mineral fibers manufacturing (OR=2.49, 95% CI=1.01, 6.12 at 3 km) and organic chemical industry facilities (OR=2.54, 95% CI=1.10, 5.90 at 2 km)

PFAS, per- and polyfluoroalkyl substances; PFOS, perfluorooctane sulfonic acid; PM_2.5_, particulate matter 2.5.

A larger case-control study conducted in California (1998–2011) looked at 221 cases of unilateral and 114 cases of bilateral retinoblastoma. These were analyzed for exposure to any of 132 pesticides using the California Cancer Registry and California Pesticide Use Reports. Cases were considered exposed if the address on the birth certificate was within 4 km of pesticide application. This group found an increased risk associated with exposure to the insecticide acephate (OR=1.70, 95% CI=1.20, 4.21) or the herbicide bromacil (OR=1.87, 95% CI=1.07, 3.26) but a decreased risk associated with exposure to the insecticide hydramethylnon (OR=0.59, 95% CI=0.35, 0.98).^35^ However, there were flaws in the methodology of this study. They used geocoding of the address at diagnosis to examine proximity to known applicator sites, acknowledging that only 54% of addresses were geocoded to the exact address, a known issue with trying to geocode in rural areas where home addresses may begin with Rural Route 1. This lack of precision could significantly impact which children were considered to have lived within the range to be exposed. Another recent study examined proximity to vineyards in France as a proxy for pesticide exposure and found no increased risk for retinoblastoma.^36^ Likewise, several groups examined whether the children of pesticide applicators had a higher risk and found no increased incidence.^37^^,^^38^ Whether pesticides are definitively involved in retinoblastoma risk is difficult to determine owing to the lack of consensus among studies and recurrent methodologic limitations (e.g., no proposed exposure pathway). More research is needed to clarify this potential connection.

There has been recent interest in the per- and polyfluoroalkyl substances (PFAS) family of chemicals and their potential role in various cancers. A recent large population-based case-control study in California examined several PFAS molecules in neonatal blood spots to determine if IQR increases in PFAS concentration correlated with an increased risk of childhood cancer.^39^ This study included 497 cases of retinoblastoma between 1983 and 2011. The authors found that infants born with above-mean perfluorooctane sulfonic acid had an increased risk of unilateral retinoblastoma (AOR=1.42, 95% CI=1.03, 1.97) and an elevated but not significant risk of bilateral retinoblastoma (AOR=1.29, 95% CI=1.00, 1.67). A nonsignificant but elevated risk was also seen for perfluorooctanoic acid. PFAS are known to pass through the placenta from mother to child, making this a plausible source of exposure.

Traffic-related air pollution has also been studied in relation to retinoblastoma; again, the results were inconsistent. Heck and colleagues^40^ modeled particulate matter 2.5 levels and traffic density within 500 m of the address on the birth certificate. One model was significant for an increased risk of unilateral retinoblastoma on the basis of particulate matter 2.5, but the other was not, and neither was significant for traffic density.^40^ Kumar et al.^41^ also looked at proximity within 500 m to major roadways in Texas (with a major roadway being defined as an interstate, state, county, or toll road highway). They found a statistically significant increased risk for unilateral retinoblastoma with every kilometer closer to the roadway where the mother lived (AOR=2.7, 95% CI=1.28, 5.15).^41^ Others, however, arrived at different conclusions. García-Pérez and colleagues^42^ looked at urban areas (as a proxy for traffic pollution) and found no increased risk there compared with rural areas. The same result was seen by Omidakhsh et al.^43^ Ghosh and colleagues^44^ found an increased risk for bilateral but not for unilateral retinoblastoma associated with increased third-trimester air pollutants, even though bilateral retinoblastoma is most often associated with paternal exposures before conception. More research is needed to clarify the potential role of vehicular air pollution in retinoblastoma development.

Heck et al.^45^ further examined air pollution using data from the California Air Resources Board Air Toxics program. Data from monitoring stations across the state sampled air quality using 24-hour samples from each monitor that were collected every 12 days. Heck’s group analyzed those samples identified by the International Agency for Research on Cancer as confirmed, plausible, or possible carcinogens. These were then correlated with the residential addresses of patients with retinoblastoma as listed on their birth certificates to estimate exposure. They found an increased risk associated with exposure during the perinatal period to styrene (OR=1.64, 95% CI=1.12, 2.39), ortho-xylene (OR=1.64, 95% CI=1.03, 2.61), and toluene (OR=1.79, 95% CI=1.10, 2.92), all components of vehicular exhaust. They also found an increased risk associated with chloroform (OR=1.53, 95% CI=1.10, 2.12), formaldehyde (OR=1.42, 95% CI=1.08, 1.88), total chromium (but not hexavalent chromium) (OR=1.34, 95% CI=1.01, 1.77), and acetaldehyde (OR=1.62, 95% CI=1.06, 2.48). However, Heck’s team was not able to adjust for smoking status owing to a lack of data, relied on the address on the birth certificate to assume continued residence, and acknowledged that the multiple comparisons in the study could have led to false positives. In addition, the air pollutants from gasoline changed during the study period, so the initial study design might not have been as useful by the end. Using similar methodology but with data from the Toxic Release Inventory, Chen’s group examined proximity to smokestacks but found no relation to retinoblastoma risk for exposure to lead, nickel, or cobalt^46^ or industrial solvents.^47^

Correlating disease incidence to air quality is difficult. In the case of the California Air Toxics program,^45^ insufficient data reduced the list of air toxics under study by almost half. Many assumptions must be made, including that the child in question is still living near the source of air pollution when critical exposures to retinoblastoma occur. In addition, air monitoring stations are located across California but are more likely to be located in urban areas. Point estimates taken are assumed to be average exposure measurements, when in fact they may be higher or lower than measured. Given that results are mixed regarding the possible connection between poor air quality and retinoblastoma incidence, more studies are needed to elucidate the relationship between these.

Garcia-Perez’s group looked at proximity to various industrial factories in their analysis. They found an increased risk near sites using glass or mineral fibers (OR=2.49, 95% CI=1.01, 6.12) or near organic chemical industries (OR=2.54, 95% CI=1.10, 5.90).^42^ When looking at specific chemicals, they showed associations with organotin compounds (OR=2.20, 95% CI=1.04, 4.66), tetrachloromethane (OR=2.54, 95% CI=1.01, 6.40), toluene (OR=3.18, 95% CI=1.29, 7.82), and trichloroethylene (OR=5.98, 95% CI=1.62, 22.04).^42^ However, many compounds and industries were compared at inconsistent distances (2 km, 3 km, 4 km, and 5 km), and only the significant distances were reported. It is possible that these results were reached by chance owing to the large number of comparisons made.

Because sunlight is taken in through the eye, several groups have looked at differences in sunlight exposure as a possible risk factor. One older study, looking at cases diagnosed between 1969 and 1987, attempted to estimate the annual ambient dose of ultraviolet B on the basis of proximity to the tropics, the peak zone of ultraviolet B exposure on earth. It reported significant increases in unilateral (but not bilateral) retinoblastoma nearer the tropics, with decreased risk further away.^48^ However, the authors admitted that most cases that they examined were not reported as unilateral or bilateral. The current understanding is that there are no geographic differences in retinoblastoma incidence. Orjuela-Grimm and colleagues^49^ also recently looked at sun exposure by estimating weekly sun exposure on the basis of residential elevation and the reported use of sun coverings on the child’s eyes. They found that cases received less sun exposure than controls in every age group studied and that sun exposure was also associated with lower levels of advanced disease.^49^ Because of these inconsistencies in results, the potential role of sunlight is thus not clear at this time.

### Occupational Risk Factors

Retinoblastoma risk associated with parental employment in a variety of industries has been considered (Table 2). A study from 1984 found that there were no differences in retinoblastoma incidence among children with parents working with ionizing radiation, but there was an increased risk among children whose fathers were radio and TV repairmen.^50^ Another study of the offspring of atomic bomb survivors found no increased risk of overall malignancies in their offspring.^51^ A large case-control study later examined exposure to medical radiation. Although they found no significant difference in unilateral retinoblastoma risk associated with maternal medical radiation, they did see an increased risk associated with upper gastrointestinal (OR=1.9, 95% CI=1.1, 3.2) and lower gastrointestinal (OR=6.9, 95% CI=2.9, 16.4) scans prior to conception.^52^

**Table 2:** Select Studies Investigating Occupational Exposures and Retinoblastoma Incidence

Occupation/occupational exposure	Study, year, location	Study design and sample size	Confounders	Findings
Farming	Bunin et al.,^56^ 1990, U.S.	Case-control, 201 cases (67 sporadic heritable, 115 nonheritable)	Other occupational risk factors, nonoccupational risk factors	Increased risk for nonheritable retinoblastoma if maternal grandfather worked in farming (OR=10.0, 95% CI=1.4, 433)
Metal working	MacCarthy et al.,^55^ 2009, Great Britain	Case-control; 1,318 cases; and 1,318 controls	Standard region, period of birth	Increased risk of nonheritable retinoblastoma with paternal occupational exposure to oil mists in metal working (OR=1.85, 95% CI=1.05, 3.36)
	Bunin et al.,^56^ 1990, U.S.	Case-control, 201 cases (67 sporadic heritable, 115 nonheritable)	Other occupational risk factors, nonoccupational risk factors	Increased risk for sporadic heritable retionblastoma with paternal employment in the metal industry (OR=Infinity, 95% CI=1.4, infinity)
Military	Bunin et al.,^56^ 1990, U.S.	Case-control, 201 cases (67 sporadic heritable, 115 nonheritable)	Other occupational risk factors, nonoccupational risk factors	Increased risk for sporadic heritable retinoblastoma with paternal employment in the military (OR=2.8, 95% CI=1.1, 8.8)
Painting and printing industries	Volk et al.,^53^ 2019, Denmark	Case-control, 8 cases (all CNS cancers), and 59 controls	SES, parental age at birth of index child, birth order, parity, maternal smoking status	Increased risk of CNS cancers associated with paternal occupation in the printing industries (OR=2.09, 95% CI=1.17, 3.75)
	Omidakhsh et al.,^57^ 2018, U.S.	Case-control, 187 unilateral cases, 95 bilateral cases, and 155 controls	Paternal age, smoking status, race/ethnicity, income, educational attainment	Increased risk of bilateral retinoblastoma with paternal occupational exposure to paints (OR=8.76, 95% CI=1.32, 58.09)
Radiation	Hicks et al.,^50^ 1984, Texas	Case-control, 298 paternal/298 maternal cases and 924 male/945 female controls	Not reported	Increased risk for children whose fathers were radio or TV repairmen (*n*=2, OR=31.44, 95% CI=4.80, 205.91)
Welding/machining	Bunin et al.,^56^ 1990, U.S.	Case-control, 201 cases (67 sporadic heritable, 115 nonheritable)	Other occupational risk factors, nonoccupational risk factors	Increased risk for nonheritable retinoblastoma associated with a job cluster that included welders and machinists (OR=4.0, 95% CI=1.1, 22.1)
Wood dust	Volk et al.,^54^ 2020, Denmark	Case-control, 11 cases and 221 controls	SES, maternal smoking	No increased risk of retinoblastoma with maternal occupational exposure to wood dust (OR=1.32, 95% CI=0.70, 2.48)

CNS, central nervous system.

No increase was found with parental employment in the painting or printing industries^53^ or with occupational exposure to wood or paper dust.^54^ However, increased risks were seen with paternal exposure to oil mists in metal working (OR=1.85, 95% CI=1.05, 3.36),^55^ in manufacturing industry (OR=1.90, 95% CI=1.30, 2.77),^43^ in the food and drink industry (OR=2.14, 95% CI=1.09, 4.21),^43^ and in welders and machinists.^56^ Maternal exposure to paints (for bilateral cases) (OR=8.76, 95% CI=1.32, 58.09)^57^ or exposure to 1 of 9 different agents that included pesticides, paints, volatile organic compounds, polycyclic aromatic hydrocarbons, or ionizing radiation led to an increased risk (OR=5.25, 95% CI=1.14, 24.16).^57^ Interestingly, whereas Fear et al.^58^ saw no increased risk of retinoblastoma associated with agricultural employment, others saw different results. Bunin’s group showed a significant association between the maternal grandfather’s work in farming and the risk of retinoblastoma in grandchildren (OR=10.0, 95% CI=1.4, 433; *p*=0.02).^56^ Because a woman’s eggs are formed during her own embryonic development, it is possible that these could be affected very early by exposures years to decades prior to the conception of her child. This could prove to be a fruitful area of research, because most studies have focused only on exposures during pregnancy or the year directly preceding it.

### Strengths, Limitations, and Future Directions

This review examined environmental and occupational risk factors for retinoblastoma as well as other risk factors that may be related to certain environments or occupations, such as exposure to radiation. However, this review was intended to inform a cancer cluster investigation rather than provide an exhaustive review of the literature. It is possible that studies relating to other risk factors may have been overlooked that would have been captured using a systematic review strategy. It is intended to inform the state of current knowledge regarding retinoblastoma risk factors and is not intended to examine causality. This review also only included papers published in English. Furthermore, because this review focused on the epidemiology of retinoblastoma in humans, potentially important information found during animal research was not considered.

Retinoblastoma research studies are difficult owing to the rarity of this form of cancer, with fewer than a dozen cases for every million children. For this reason, many studies fail to find statistically significant differences between comparison groups. Furthermore, with few methods available to measure past exposures, many studies have relied on surveys of parents several years later, a method that is susceptible to recall bias. These problems are inherent in the study of rare conditions and make determining risk factors challenging.

In the absence of risk factors that could be used for primary prevention, continued screening for retinoblastoma during well-child visits remains critical. Retinoblastoma survival rates depend heavily on when this cancer is diagnosed, so early detection is key to improving overall survival. Maximizing adherence to routine red reflex testing, as recommended by the American Academy of Pediatrics, is thus vitally important. Also important is emphasizing smoking cessation during pregnancy, because this appears to be a risk factor for retinoblastoma as well as several other pediatric cancers. Although retinoblastoma incidence might not currently be alterable, increasing survival can be achieved with public health support for prenatal and early childhood health care.

## CONCLUSIONS

DHS’s attempt to investigate a potential cluster of retinoblastoma cases in Wisconsin was unsuccessful owing to the small number of studies investigating links between the incidence of this cancer and environmental or occupational risk factors. This is a common problem within cancer cluster investigations involving rare cancers, particularly those in children, where cancer itself is a rare event. Although some studies have shown potential associations (such as those examining the impact of pesticides), these results need to be corroborated. Exposure pathways between suspected environmental agents and the cells at risk also need to be elucidated. Further research is needed before it can be determined whether there are environmental or occupational risk factors for sporadic retinoblastoma and, if so, to what extent they influence incidence or disease progression.
